# The association between enlarged perivascular spaces and muscle sympathetic nerve activity in normotensive and hypertensive humans

**DOI:** 10.1007/s10286-025-01160-6

**Published:** 2025-10-11

**Authors:** Donggyu Rim, William Pham, Rania Fatouleh, Annemarie Hennessy, Markus Schlaich, Luke A. Henderson, Vaughan G. Macefield

**Affiliations:** 1 Department of Neuroscience, Monash University, Level 6, 99 Commercial Road, Melbourne, VIC 3004 Australia; 2 Baker Heart and Diabetes Institute, Melbourne, VIC Australia; 3 Baker Department of Cardiometabolic Health, University of Melbourne, Melbourne, VIC Australia; 4 Faculty of Medicine and Health, University of Sydney, Sydney, NSW Australia; 5 Heart Research Institute, University of Sydney, Sydney, NSW Australia; 6 Dobney Hypertension Centre, Medical School – Royal Perth Hospital Unit and RPH Research Foundation, University of Western Australia, Perth, WA Australia; 7 Department of Nephrology and Cardiology, Royal Perth Hospital, Perth, WA Australia; 8 Faculty of Medicine and Health, School of Medical Sciences (Neuroscience), Brain and Mind Centre, University of Sydney, Sydney, NSW Australia

**Keywords:** Glymphatic, Magnetic resonance imaging, Muscle sympathetic nerve activity, Perivascular spaces

## Abstract

**Purpose:**

Hypertension is characterised by both enlarged perivascular spaces (ePVS) and chronically elevated resting sympathetic outflow. ePVS is associated with heart rate variability, suggesting links to autonomic outflow; however, heart rate variability offers limited information on sympathetic nerve activity. Here, we assessed whether ePVS are associated with muscle sympathetic nerve activity (MSNA) in 25 hypertensive patients and 50 healthy normotensive adults.

**Methods:**

T1-weighted MRI anatomical brain images were analysed for ePVS using a deep learning-based segmentation algorithm (nnU-Net). Spontaneous bursts of MSNA were recorded from the right common peroneal nerve via a tungsten microelectrode immediately before the MRI scan in a supine position. A backward regression analysis was conducted to test the relationship between ePVS and MSNA.

**Results:**

Significant associations were found between MSNA and ePVS in the white matter (*β* = 1.02, *p* = 0.007), basal ganglia (*β* = 0.43, *p* = 0.001), and hippocampus (*β* = 0.24, *p* = 0.010) in healthy normotensive adults. Similar associations were observed in individuals with hypertension. Notably, the association between MSNA and midbrain ePVS cluster was only observed in the hypertensive group (*β* = 0.41, *p* = 0.005).

**Conclusion:**

ePVS were associated with MSNA in both normotensive and hypertensive patients. These findings warrant further research into the causal relationship between MSNA and ePVS and highlight the potential for ePVS as a neuroimaging biomarker for sympathetic nerve activity.

**Supplementary Information:**

The online version contains supplementary material available at 10.1007/s10286-025-01160-6.

## Introduction

Perivascular spaces (PVS) are fluid-filled spaces surrounding penetrating cerebral arterioles, formed between the vascular smooth muscle basement membrane and the ensheathing astrocyte end-feet [1]. Dysfunction of PVS, indicated by dilated or enlarged PVS (ePVS), can be visualised on magnetic resonance imaging (MRI) scans in specific brain regions including basal ganglia, centrum semiovale, midbrain and hippocampus [1]. ePVS is an early marker of cerebral small vessel disease (CSVD) [2] and may contribute to neuroinflammation [3] and disrupted waste clearance through the glymphatic system [4], and is associated with hypertension [5–7].

Previous studies have revealed an association between ePVS and heart rate variability (HRV) [8, 9], which indicates ePVS may be linked to autonomic nervous system outflow. However, these studies have not examined individuals with hypertension, despite hypertension being strongly associated with ePVS [5–7, 10, 11] and autonomic dysfunction [12–14]. In addition, there are controversies regarding the utility of HRV as a marker for autonomic activity, as it is an indirect measurement of the cardiac autonomic activity [15]. Moreover, the low-frequency component of HRV may not accurately represent sympathetic outflow in humans as a result of crossover from parasympathetic and respiratory signals that overlap in the frequency band [16–18]. Microneurography, the gold standard technique, directly records sympathetic outflow from the postganglionic axons innervating muscle vascular bed, known as muscle sympathetic nerve activity (MSNA) [19–22].

MSNA is generated in the brain and contributes to the beat-to-beat regulation of blood pressure [23, 24]. Meta-analysis has shown elevated MSNA across all stages of hypertension irrespective of treatment status [14]. Notably, antihypertensive treatments often fail to reduce MSNA to normative levels, suggesting that current therapies do not adequately address the underlying mechanisms driving sympathetic overactivity [25]. Sympathetic outflow is influenced by neuroinflammation [26–28], astrocytic function [29–32], and brain structure [33, 34]. These factors are not only altered in hypertension but have also been independently associated with ePVS [3, 35–37]. Given that neuroinflammation, astrocytic dysfunction, and structural brain changes have all been independently linked to both elevated MSNA and ePVS in previous studies, it is plausible that these two phenomena may be related. This hypothesis warrants further investigation, as it may reflect shared underlying pathophysiology. A better understanding of this link could help explain persistent sympathetic activation in hypertension and could help lower residual cardiovascular risk beyond conventional blood pressure control [25].

In this study, we aimed to assess whether MSNA is associated with MRI-visible ePVS in both normotensive and hypertensive adults. We hypothesised that ePVS are associated with MSNA in the hypertensive but not in the normotensive group, as hypertension is associated with elevated sympathetic outflow and ePVS.

## Methods

### Study design

This cross-sectional study was approved by the Western Sydney University Human Research Ethics Committee (HREC approval H11462) and endorsed by Governance at the Baker Heart and Diabetes Institute. Informed written consent was obtained from all participants and conformed to the Declaration of Helsinki. All participants refrained from caffeinated drinks, vigorous exercise and nicotine from the morning of the experiment until completion. All experiments were conducted at 12 PM.

### Participants

A total of 25 hypertensive patients and 50 healthy normotensive participants were recruited for this study. Participants previously diagnosed with hypertension and currently using peripheral and centrally acting antihypertensive medications (Table S4), and participants who had a seated average systolic BP ≥ 130 mmHg and diastolic BP ≥ 80 mmHg were classified as hypertensive for this study, according to the American College of Cardiology/American Heart Association guideline [38]. Normotensive participants were defined as subjects with a seated average systolic BP < 130 mmHg and diastolic BP < 80 mmHg, without any history of diseases, or taking any antihypertensive medications. Participants were excluded if they (i) had a previous history of cardiovascular disease such as heart failure or stroke; (ii) had any types of neurodegenerative disease such as Alzheimer’s, Parkinson’s disease or amyotrophic lateral sclerosis; (iii) had conditions contraindicated for MRI scanning, such as having cardiac pacemakers, metallic implants, or aneurysm clips.

### Data acquisition

Blood pressure measurement was taken after a 5-min resting period in a seated position with an automated sphygmomanometer (HEM7141, Omron, Japan). An average of three blood pressure measurements, taken with a 1-min rest period in between the measurements, was reported. The participants refrained from talking, had feet flat on the floor, and their backs were supported during the measurements.

Microneurography was performed with participants lying supine on an MRI bed. MSNA was obtained using the standard recording protocol established in our lab [39]. In addition to microneurography, electrocardiogram (ECG, 2 kHz, three Ag/AgCl surface electrodes; Covidien, Ireland), and continuous non-invasive blood pressure recording were obtained from finger cuffs (NOVA, Finapres Medical System BV, Netherlands). MSNA bursts were counted using the Cyclic Measurements peak detection feature of LabChart (LabChart for Macintosh, v7.2.5; ADInstruments). The numbers of bursts in 1 min (burst frequency, BF) and 100 heartbeats (burst incidence, BI) were calculated for each participant.

### Imaging acquisition

Twenty-eight T1-weighted anatomical images of the brain were acquired using a Siemens Magnetom 3 T (64-channel SENSE coil, repetition time [TR] = 2300 ms, echo time [TE] = 2.49 ms, flip angle = 8°, 192 sagittal slices, voxel size = 0.9 mm isotropic), while 47 T1-weighted anatomical scans were obtained using a Philips Achieva 3 T (32-channel SENSE head coil TR = 5600 ms, TE = 2.5 ms, flip angle = 8°, 200 sagittal slices, voxel size = 0.87 mm isotropic).

### Imaging processing

**Fig. 1:**
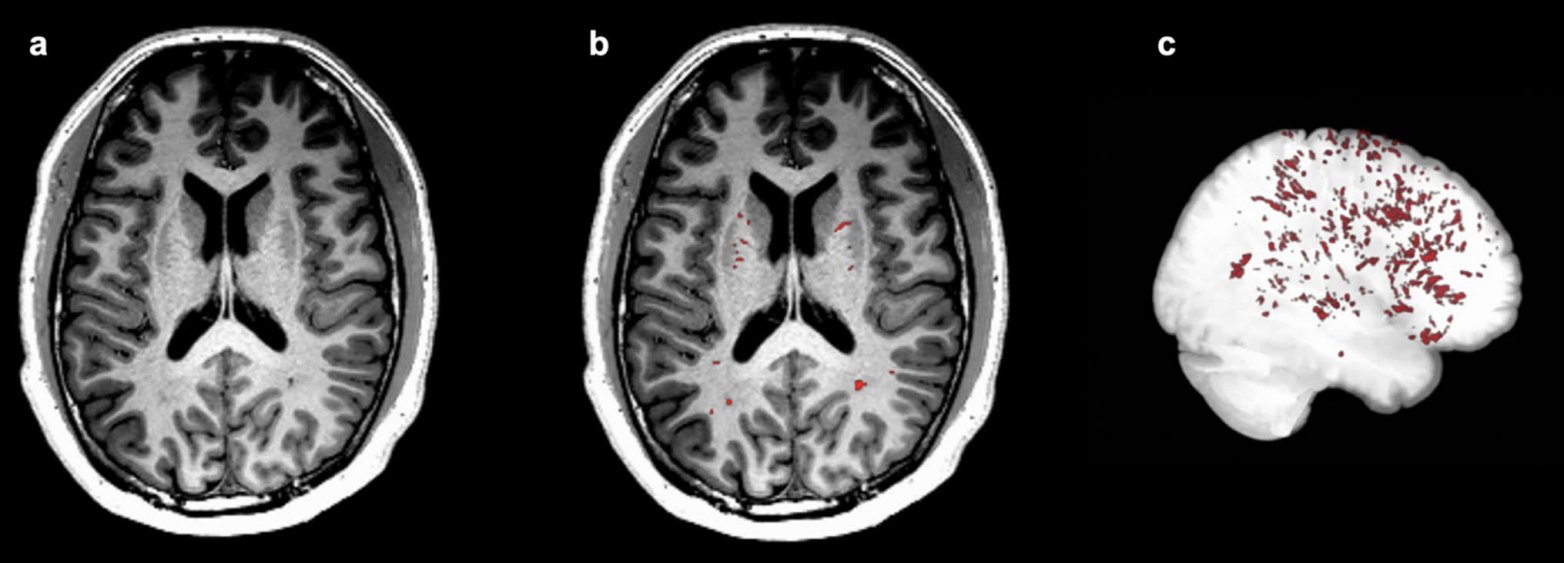
Perivascular spaces segmentation example using nnU-Net, deep learning-based segmentation methods. **a** Axial slice of T1-weighted MRI before segmentation. **b** Representation of segmented perivascular spaces on the T1-weighted MRI indicated by red. Labelling of perivascular spaces appears in the basal ganglia and white matter regions. **c** 3D-rendered perivascular spaces segmentation across the whole brain

ePVS were identified using a deep learning-based segmentation algorithm (nnU-Net residual encoder) from the T1-weighted anatomical scans. nnU-net is a self-configuring deep-learning-based segmentation method that incorporates preprocessing, network architecture, training, and post-processing for biomedical images [40]. The U-Net architecture relies on convolutional filters arranged in a U-shaped configuration to extract image features of interest. Upon visual inspection, the model can accurately segment PVS voxels in the white matter (centrum semiovale), basal ganglia (BG), hippocampus (HP), and midbrain (MB). The region of interest analysis of these four regions was performed as MRI-visible ePVS are limited only to these four characterised areas of the brain [1]. Subsequently, it was used to predict PVS in this dataset (*n* = 75) as shown in Fig. 1. All identified MRI-PVS were considered enlarged as the model was trained with manually labelled ePVS reviewed by a radiologist. The total number of distinct ePVS voxel clusters was represented as “ePVS cluster”, and the total PVS voxels in all clusters multiplied by the voxel size of the image was represented as “ePVS volume”. The algorithm was further optimised by using the sparse annotation method, target spacing optimisation, image preprocessing optimisation using non-local means filtering (NLMF) and adaptive histogram equalisation (AHE), and semi-supervised learning for T1-weighted anatomical images [41]. Total intracranial volume (TIV), total grey matter (GM) volume, and total white matter (WM) volume were obtained with a computational anatomy toolbox (CAT12) [42] for statistical parametric mapping (SPM) version 12.

### Statistical analysis

Statistical analyses were performed using GraphPad Prism (v9.5.1 for Macintosh, San Diego, CA, USA) and IBM SPSS Statistics (v26.0, Armonk, NY, USA). Participant characteristics were assessed for normality using the Shapiro–Wilk test. Continuous variables are reported as mean ± standard error of the means, as well as 95% confidence interval (CI). Categorical variables are shown as percentages. We conducted a correlation analysis using a Spearman nonparametric correlation matrix with GraphPad Prism to determine the association between ePVS (cluster or volume) and MSNA (incidence or frequency) as well as other participant characteristics such as age, sex, and haemodynamic measurements. For the ePVS regions that showed a significant correlation with MSNA, additional group-level simple linear regressions were conducted between the ePVS and each MSNA metric (BI and BF). A backward multiple regression analysis was conducted to assess the relationship between ePVS and MSNA after adjusting for confounding factors such as age, sex, mean blood pressure, medication status, TIV, total GM volume, total WM volume, and scanner. Moreover, additional backward multiple regression analysis was conducted to assess the relationship between ePVS and haemodynamic measurements such as the mean blood pressure. The criterion for the backward elimination method was set at a probability of F-to-remove greater than or equal to 0.1.

## Results

A total of 75 participants were included in the study. Twenty-five participants were hypertensive patients, and 50 participants were normotensive. The two groups had no statistically significant differences in age. However, significant differences were observed in the male-to-female ratio between the groups. As expected, all haemodynamic measurements were elevated in the hypertension group compared to the normotensive group. The standard metrics of MSNA measurements were elevated in the hypertensive group compared to the controls, reflecting the increases in central sympathetic drive to the peripheral blood vessels with hypertension. Overall, the number of WM ePVS in the hypertension group was reduced compared to the normotensive group, but the volume of WM ePVS was not significantly different between the groups. Conversely, there were no statistically significant differences in ePVS between the two groups for the BG, HP, and MB. There was no statistically significant difference in the TIV between the groups, but the hypertensive group had reduced total GM volume and total WM volume. Data are presented in Table 1.

**Table 1:** Participant characteristics

Participant characteristics	Hypertension	Normotension	*p* value	95% CI
No.	25	50		
Age (years)	40.8 ± 4.1	34.7 ± 2.1	0.1463	[− 2.19, 14.46]
Sex			0.0080	
Male (%)	20 (80%)	24 (48%)		
Female (%)	5 (20%)	26 (52%)		
Haemodynamic measures				
MBP (mmHg)	103 ± 2.1	86 ± 1.2		
SBP (mmHg)	148 ± 4.0	121 ± 1.0		
DBP (mmHg)	82 ± 2.0	68 ± 1.1		
PP (mmHg)	57 ± 6.8	42 ± 1.9		
MSNA				
BF (bursts/min)	28.5 ± 3.3	20.9 ± 1.5	0.0167	[− 13.91, − 1.428]
BI (bursts/100 heartbeats)	48.2 ± 5.8	33.3 ± 2.3	0.0056	[− 25.33, − 4.492]
White matter ePVS				
No. clusters	635.2 ± 12.33	707.4 ± 10.81	< 0.0001	[37.12, 107.4]
Volume (mm^3^)	1857 ± 244.3	1536 ± 126.1	0.3621	[− 814.9, 173.3]
Basal ganglia ePVS				
No. clusters	31.96 ± 2.0	34.96 ± 1.3	0.1979	[− 1.60, 7.60]
Volume (mm^3^)	567.0 ± 47.3	528.8 ± 20.6	0.9887	[− 126.5, 50.08]
Hippocampus ePVS				
No. clusters	1.44 ± 0.34	1.92 ± 0.27	0.3191	[− 0.42, 1.38]
Volume (mm^3^)	4.73 ± 1.36	6.80 ± 1.23	0.2709	[− 1.89, 6.05]
Midbrain ePVS				
No. clusters	2.80 ± 0.45	3.46 ± 0.29	0.0744	[− 0.36, 1.68]
Volume (mm^3^)	10.02 ± 1.53	11.70 ± 1.60	0.8692	[− 3.34, 6.69]
Total intracranial volume (cm^3^)	1460 ± 26.24	1527 ± 21.43	0.0632	[− 3.79, 137.9]
Grey matter volume (cm^3^)	635.2 ± 12.33	707.4 ± 10.81	< 0.0001	[37.12, 107.4]
White matter volume (cm^3^)	517.5 ± 9.17	553.2 ± 9.69	0.0233	[5.45, 65.99]

Data presented as the mean, ± standard error of the mean, 95% confidence interval relating to the difference in each characteristic between the hypertension and normotension groups

*BMI* body mass index, *HR* heart rate, *MBP* mean blood pressure, *SBP* systolic blood pressure, *DBP* diastolic blood pressure, *PP* pulse pressure, *MSNA* muscle sympathetic nerve activity, *BF* burst frequency, *BI* burst incidence, *ePVS* enlarged perivascular spaces

The association between MSNA and ePVS, and its relationships with other participant characteristics were first assessed using a Spearman correlation matrix (Fig. S1). MSNA BF and BI were positively associated with ePVS volume in the BG, as well as with the MB. Interestingly, the BG, MB, and WM ePVS volumes were positively associated with age. Moreover, MSNA BF and BI showed a positive association with age. With regard to the haemodynamic measures, MSNA BI was positively correlated with mean blood pressure (MBP) as well as with diastolic blood pressure (DBP) but was negatively correlated with pulse pressure (PP). MSNA BF was not associated with MBP but was positively associated with DBP and negatively associated with PP. The number of ePVS clusters in the BG and MB showed negative correlations with DBP. The WM ePVS cluster was positively correlated with PP and sex. Medication use was positively associated with MSNA BF, BI, WM ePVS volume, and BG ePVS volume.

The group-level associations between MSNA and ePVS volume were assessed with simple linear regression analysis (Fig. S2). We observed a significant positive correlation between MSNA BF and BI with BG ePVS volume in both the normotensive group and the hypertensive group. Interestingly, the correlation between MSNA BF and BI with midbrain ePVS volume was only observed in the normotensive group. No correlation was observed between WM or HP ePVS and MSNA.

Multiple linear regression analyses of the MSNA as a dependent variable were performed to determine if ePVS was associated with MSNA when adjusted for confounding factors such as age, sex, BP, and TIV. The results of the pooled analysis (*n* = 75:* n*_hypertensive_ = 25,* n*_normotensive_ = 50) (Table 2) revealed that MSNA BI was associated with sex, medication status, the number of ePVS clusters in MB, TIV, total WM volume, and scanner with the adjusted* R*-squared of 60%. Moreover, MSNA BF was associated with sex, volume of WM ePVS, number of WM ePVS clusters, as well as the volumes of BG ePVS and MB ePVS, and scanner with the adjusted* R*-squared of 56%.

**Table 2:** Backward multiple linear regression analysis with muscle sympathetic nerve activity as dependent variable (*n* = 75)

	Burst incidence		Burst frequency	
	*β*	*p*	*β*	*p*
Age (years)	–	–	–	–
Sex	0.195	0.019	0.298	0.001
Hypertension status	–	–	–	–
Medication status	0.298	0.002	–	–
Mean BP (mmHg)	–	–	–	–
WM ePVS volume (mm^3^)	–	–	0.961	0.005
WM ePVS cluster	–	–	-1.089	0.003
BG ePVS volume (mm^3^)	–	–	0.283	0.005
BG ePVS cluster	–	–	–	–
HP ePVS volume (mm^3^)	–	–	–	–
HP ePVS cluster	–	–	–	–
MB ePVS volume (mm^3^)	–	–	0.227	0.009
MB ePVS cluster	0.277	0.001	–	–
TIV	− 0.374	0.033		
GM	–	–		
WM	0.459	0.009		
Scanner	− 0.570	< 0.001	− 0.382	0.002
Adjusted *R*^2^	0.60		0.56	

*BP* blood pressure, *PVS* perivascular space, *ePVS* enlarged perivascular space, *WM* white matter, *BG* basal ganglia, *HP* hippocampus, *TIV* total intracranial volume, *GM* grey matter, *β* standardised coefficient beta

The subgroup regression analysis for the hypertensive group (*n* = 25, Table 3) revealed that MSNA BI was associated with age, BG ePVS volume, HCP ePVS volume, number of MB ePVS cluster and TIV, with the adjusted* R*-squared of 69%. Moreover, MSNA BF in the hypertensive group was associated with WM ePVS volume, number of WM and MB ePVS clusters, TIV, and total WM volume, with the adjusted* R-*squared of 65%.

**Table 3:** Backward multiple linear regression analysis with muscle sympathetic nerve activity as dependent variable (*n* = 25 hypertensive patients)

	Burst incidence		Burst frequency	
	*β*	*p*	*β*	*p*
Age (years)	0.730	< 0.001	–	–
Sex	–	–	–	–
Mean BP (mmHg)	–	–	–	–
Medication status	–	–	–	–
WM ePVS volume (mm^3^)	–	–	2.409	< 0.001
WM ePVS cluster	–	–	− 2.298	< 0.001
BG ePVS volume (mm^3^)	− 0.382	0.028	–	–
BG ePVS cluster	–	–	–	–
HP ePVS volume (mm^3^)	0.668	0.045	–	–
HP ePVS cluster	–	–	–	–
MB ePVS volume (mm^3^)	–	–	–	–
MB ePVS cluster	0.316	0.022	0.409	0.005
TIV	− 0.328	0.012	− 0.565	0.034
GM	–	–	–	–
WM	–	–	0.541	0.034
Scanner	–	–	–	–
Adjusted* R*^2^	0.69		0.65	

*BP* blood pressure, *PVS* perivascular space, *ePVS* enlarged perivascular space, *WM* white matter, *BG* basal ganglia, *HP* hippocampus, *TIV* total intracranial volume, *GM* grey matter, *β* standardised coefficient beta

The subgroup regression analysis of the normotensive group (*n* = 50, Table 4) showed that MSNA BI were associated with age, sex, number of WM and HP ePVS clusters, BG ePVS volume, and scanner with the adjusted* R*-squared of 62%. Also, MSNA BF in the normotensive group was positively correlated to age, sex, WM and BG ePVS volume, and negatively correlated to number of WM ePVS clusters, with the adjusted* R*-squared of 49%.

**Table 4:** Backward multiple linear regression analysis with muscle sympathetic nerve activity as dependent variable (*n* = 50 normotensive adults)

	Burst incidence		Burst frequency	
	*β*	*p*	*β*	*p*
Age (years)	0.255	0.032	0.361	0.005
Sex	0.305	0.006	0.273	0.042
Mean BP (mmHg)	–	–	–	–
WM ePVS volume (mm^3^)	–	–	1.021	0.007
WM ePVS cluster	− 0.294	0.039	− 1.483	< 0.001
BG ePVS volume (mm^3^)	0.349	0.002	0.432	0.001
BG ePVS cluster	–	–	–	–
HP ePVS volume (mm^3^)	–	–	–	–
HP ePVS cluster	0.244	0.010	–	–
MB ePVS volume (mm^3^)	–	-	–	–
MB ePVS cluster	–	–	–	–
TIV	–	–	–	–
GM	–	–	–	–
WM	–	–	–	–
Scanner	− 0.491	< 0.001		
Adjusted* R*^2^	0.62		0.49	

*BP* blood pressure, *PVS* perivascular space, *ePVS* enlarged perivascular space, *WM* white matter, *BG* basal ganglia, *HP* hippocampus, *TIV* total intracranial volume, *GM* grey matter, *β* standardised coefficient beta

Multiple linear regression was also performed on each haemodynamic measurement as the dependent variable to determine the relationship between ePVS and haemodynamic measurements after adjusting for confounding factors. The pooled analysis of all participants revealed a positive association between the WM ePVS cluster with PP (Table S1). BG ePVS volume was negatively correlated with DBP. HP ePVS volume was positively associated with MBP, SBP, and DBP. MB ePVS volume was negatively correlated with SBP and PP. In the hypertensive patients, WM ePVS volume was negatively associated with all haemodynamic measurements and number of WM ePVS clusters was positively associated with all haemodynamic measurements (Table S2). A similar pattern was observed in BG ePVS volume, which showed a negative association with MBP and DBP, and a positive association between the number of BG ePVS clusters with MBP and DBP. HP ePVS volume was positively associated with MBP and SBP, and number of HP ePVS clusters was negatively associated with SBP and PP. MB ePVS volume showed a positive association with DBP but a negative association with PP. Conversely, the number of MB ePVS clusters showed negative association with DBP but positive association with PP. In the normotensive adults, SBP was negatively associated with the BG ePVS volume, TIV, and positively associated with total WM volume (Table S3).

## Discussion

Previous studies have suggested that ePVS is an emerging biomarker for a variety of disease states, including CSVD and hypertension [37, 43, 44]. How ePVS contribute to the sympathetic nervous system has remained inconclusive. We have shown, for the first time, that MRI-visible ePVS are associated with MSNA. Contrary to our hypothesis, ePVS was associated with MSNA in *both* normotensive and hypertensive adults. After adjusting for age, sex, MBP, TIV, total GM volume, and total WM volume using multiple linear regression, we observed that MSNA in the hypertensive group was associated with ePVS in the WM, BG, HP, and MB, while the MSNA in the normotensive group was associated with ePVS in the WM, BG, and HP, but not in the MB. Our finding that MB ePVS is positively associated with sympathetic nerve activity in the hypertensive group but not in the normotensive group suggests that MB ePVS are associated with the increased sympathetic vasoconstrictor drive in hypertension.

### Association between ePVS and sympathetic nerve activity

Previous studies have elucidated the association between the autonomic nervous system and ePVS using HRV measurements [8, 9]. A previous study by Zhou et al. has shown an association between HRV measures, including root mean square of successive differences of normal-normal intervals for the period of interest (rMSSD) and the ratio of low-frequency power to high-frequency power (LF/HF) with BG-ePVS severity [8]. Another study has shown an association between lower HRV measured by the standard deviation of normal-to-normal intervals (SDNN) and severe BG-ePVS [9]. However, these studies have not obtained direct recording of sympathetic nerve activity, limiting the interpretation of the relationship between the sympathetic nervous system and ePVS. Indeed, rMSSD is an estimate of vagal (parasympathetic) influence [45], and LF/HF ratios, while assumed to estimate the sympathetic and parasympathetic balance, are highly variable with measurement conditions [45] and oversimplify the non-linear, and in some cases non-reciprocal, interaction between the two autonomic nervous systems [15]. While SDNN has shown utility in stratifying cardiac risks [46, 47], both sympathetic and parasympathetic components contribute to SDNN and, therefore, have limited capacity to delineate the association between the *sympathetic nervous system* and ePVS. In light of this, our finding reveals an association between direct sympathetic outflow and ePVS in all four regions in the hypertensive patients (BG, WM, MB, HP) and BG, WM, and HP in normotensive adults. These results suggest that future work is necessary to investigate the causal relationship between ePVS and sympathetic nerve activity in health and disease.

Several mechanisms may be hypothesised to underlie the associations observed in our study. ePVS may disrupt sympathetic outflow via alteration of grey matter volumes. The midbrain, which was shown to have association with ePVS and MSNA in the hypertensive individuals from our results, contains key structures, such as the periaqueductal gray (PAG), known to modulate sympathetic nerve activity [48, 49]. Given that altered grey matter volume affects resting levels of MSNA [12, 33, 34], and ePVS is associated with changes in grey matter volume [50], the association between ePVS and MSNA is hypothesised to be related to altered grey matter volume. Moreover, another explanation for the association between ePVS and MSNA may be related to the disruption of astrocytes. In hypertension, astrocytes are perturbed [51], leading to the penetration of leukocytes into the parenchyma as well as disrupted fluid homeostasis via aquaporin 4 channel dysfunction, leading to the formation of ePVS [35]. Therefore, the ability of astrocytes to monitor changes in cerebral perfusion pressure [30] and elevate sympathetic nerve activity [52] may be compromised in hypertension. Moreover, the mechanical sheer force from the excess interstitial fluids in ePVS may contribute to astrocyte dysfunction, as interstitial fluid-mediated shear stress can alter angiotensin II signalling in the rostral ventrolateral medulla (RVLM) astrocytes and induce sympathoinhibitory effects [53]. Future studies should be conducted to investigate whether disturbed astrocytes underlie the association between ePVS and sympathetic outflow in hypertension.

However, it must be acknowledged that reverse causality may be true, given the cross-sectional nature of this study, that is sustained elevation of sympathetic nerve activity may contribute to the development of ePVS. We have recently shown that hypertension-induced grey matter changes in the brain are associated with MSNA [12]. It may be possible that grey matter disruption associated with sustained sympathetic elevation may contribute to the development of ePVS. Recently, it has been shown that MSNA is associated with cerebral blood velocity in health individuals [54]. While it is not clear whether these relationships hold in hypertensive patients, it may be hypothesised that sustained MSNA (in hypertension) may impact cerebral blood velocity and contribute to the disruption in perivascular spaces. Furthermore, sustained elevations in sympathetic nerve activity is hypothesised to contribute to elevated intracranial pressure and may lead to disrupted blood–brain barrier, which may impact perivascular spaces [55]. Further longitudinal studies are suggested to clarify the causal relationship between ePVS and MSNA in hypertension.

### Relationship between ePVS and blood pressure

We found that the hypertensive group showed a positive relationship between WM ePVS clusters with MBP, SBP, DBP and PP, which was not observed in the controls. This is consistent with the previous work showing that cumulative BP exposure was positively associated with increases in WM ePVS score [6]. Moreover, we found that WM ePVS volume was inversely correlated to MBP, SBP, DBP and PP, which may indicate that the hypertensive patients may have a higher number of ePVS clusters with smaller volumes. Indeed an intensive systolic blood pressure treatment has been shown to reduce ePVS volume [56]. Therefore, it may be possible that the antihypertensive medication use in the hypertensive group may have influenced the WM ePVS volume. Moreover, there seems to be a mechanism that may limit the association between WM ePVS and blood pressure in the normotensive group, as we did not observe a significant relationship between WM ePVS and haemodynamic measurements after adjusting for confounding factors. This may be an important consideration for further work, noting that WM ePVS, specifically at the centrum semiovale, is associated with β-amyloid in patients with Alzheimer’s disease [57].

Our results revealed no statistical differences between the normotensive and hypertensive groups in regard to total ePVS volume and cluster in the BG, HP, and MB and the ePVS volume in the WM (Table 1). However, to our surprise, the total number of ePVS clusters in WM was significantly reduced in the hypertensive group compared to the normotensive group. The finding of fewer (but larger—though not statistically significant) WM ePVS clusters in hypertensive group compared to normotensive group was unexpected, given the established increased ePVS burden typically associated with hypertension [6, 10], as well as the relationship observed between haemodynamic measurements and WM ePVS in the hypertensive cohort. Some possible reasons may be due to the significant white matter atrophy observed in the hypertensive compared to the normotensive cohort, which may have reduced the MRI-visible WM ePVS that could be detected. This hypothesis is supported by a previous study that reported that WM ePVS is associated with total intracranial volume [58]. Therefore, to adjust for this confounding factor, we undertook further analyses for TIV, total WM volume, and total GM volume in all participants.

Moreover, intensive antihypertensive treatment has been previously shown to be associated with decreased PVS volume [56]. It may be possible that antihypertensive medication use in the hypertensive cohort affected the number of WM ePVS. However, given that the ePVS in other regions were not affected, further studies are needed to confirm whether WM ePVS is particularly sensitive to antihypertensive medication use compared to other regions. Furthermore, white matter hyperintensities (WMH) often topologically overlap with the WM ePVS [59], making it difficult to accurately quantify WM ePVS with only T1-weighted anatomical images. Increased WMH in hypertension [60, 61] may have contributed to the lower quantification of WM ePVS in the hypertensive group. Further work should utilise T2-weighted imaging to delineate WMHs from WM ePVS.

### Limitations

One limitation of this study is its relatively small sample size. Neuroimaging studies often underestimate the sample size required to generate statistically robust results and should consider using more than a thousand participants [62]. However, given the invasive nature of recording sympathetic nerve activity via microneurography and the time taken to obtain suitable data, undertaking such a large study would be impractical. Another potential confounding factor is individual differences in total brain volumes (TIV, total GM volume, and total WM volume), which may have influenced the amount of PVS detected. Therefore, we have adjusted all further analyses with the individual participant TIV, total WM volume, and total GM volume, as mentioned previously.

Our investigation was a multi-site study that utilised two separate 3-T MRI scanners from different manufacturers. Although the differences in voxel size were accounted for during the process of segmentation and pre-processing of the images, differences in resolution may have affected our results. Moreover, using higher resolution scanners such as 7-T scanners may enhance resolution and may yield more accurate detection of ePVS in humans [63]. Furthermore, MRI-visible ePVS are restricted to macroscopically visible enlarged spaces, which may not represent the entire perivascular network, such as pericapillary or perivenular spaces [3, 64]. Also, it has been suggested that ePVS may be better visualised on the T2-weighted sequence than the T1-weighted sequence [65]. However, we utilised an automated ePVS detection model optimised for T1-weighted images obtained from different scanners [41], which enhances the accuracy of ePVS detection in the current dataset.

One major concern is the confounding effects of antihypertensive medication use in patients. Each antihypertensive drug may have a unique effect on sympathetic outflow. Angiotensin II receptor blockers (ARB) have been shown to reduce MSNA and the activity of RVLM neurons. As well as this, angiotensin-converting enzyme (ACE) inhibitors also have shown sympathoinhibitory effects [66]. The participants enrolled in the current study were taking a wide range of antihypertensive medications in different dosages and frequencies, which makes the interpretation limited. Four participants were on combination therapy (two or more of ARBs, ACE inhibitors, diuretics, calcium channel blockers or centrally acting antihypertensives), three participants on ARB or ACE inhibitor monotherapy, five participants with undefined peripheral antihypertensive medications and 13 untreated hypertensive patients. Despite this, the overall group sympathetic nerve activity was elevated in the hypertensive group compared to the normotensive group, reflecting an increase in sympathetic outflow in hypertension. Future larger sample studies should consider stratifying patients by medication use and the type of antihypertensive medication.

### Perspective

The long-standing limitation in applying microneurography in clinical settings relates to its time-consuming nature; the procedure ranges between 1.5 and 3 h. Therefore, it is an impractical technique in clinical settings. Our results suggest the potential use of MRI-visible ePVS as an imaging biomarker for individuals with high sympathetic activity. Moreover, the advancement of automated segmentation of ePVS allows accurate and efficient identification of ePVS acquired from T1-weighted anatomical images, which are routinely obtained in a standard imaging protocol. Such an imaging marker may assist in identifying individuals who require microneurographic assessment of sympathetic nerve activity.

## Conclusions

This study demonstrates region-specific associations between ePVS in the brain and MSNA in both healthy normotensive and hypertensive individuals. Importantly, those with hypertension also showed an additional association between ePVS and MSNA in the midbrain. These findings suggest that while ePVS may influence sympathetic nerve activity in general, midbrain involvement may be a distinctive feature of hypertension.

## Supplementary Information

Below is the link to the electronic supplementary material.

Supplementary file1 (DOCX 264 KB)

## Data Availability

Data available upon reasonable request.

## Abbreviations

ACE: Angiotensin-converting enzyme
AHE: Adaptive histogram equalisation
ARB: Angiotensin II receptor blocker
BF: Burst frequency
BG: Basal ganglia
BI: Burst incidence
BP: Blood pressure
CAT12: Computational anatomy toolbox
CI: Confidence interval
CSVD: Cerebral small vessel disease
DBP: Diastolic blood pressure
ECG: Electrocardiogram
ePVS: Enlarged perivascular spaces
GM: Grey matter
HP: Hippocampus
HRV: Heart rate variability
LF/HF: Low-frequency power to high-frequency power
MB: Midbrain
MBP: Mean blood pressure
MRI: Magnetic resonance imaging
MSNA: Muscle sympathetic nerve activity
NLMF: Non-local means filtering
PAG: Periaqueductal gray
PP: Pulse pressure
PVS: Perivascular spaces
RMS: Root mean squared
rMSSD: Root mean square of successive differences of normal-normal intervals
RVLM: Rostral ventrolateral medulla
SBP: Systolic blood pressure
SDNN: Standard deviation of normal-to-normal intervals
SPM: Statistical parametric mapping
TIV: Total intracranial volume
WM: White matter
