# Supplementary material for: The association between enlarged perivascular spaces and muscle sympathetic nerve activity in normotensive and hypertensive humans
Source: Clin Auton Res. 2025 Oct 11;35(6):735–45. doi: 10.1007/s10286-025-01160-6 (PMC12664836; doi:10.1007/s10286-025-01160-6)
Supplement: Supplementary file 1 — Supplementary file1 (DOCX 264 KB) [file 10286_2025_1160_MOESM1_ESM.docx]

**SUPPLMENTARY MATERIAL**

**_Figure S1._** _Correlation matrix showing associations of MSNA, PVS, age, haemodynamic measurements, sex, medication use and scanner. n=75 (pooled data from: 25 hypertensives and 50 normotensive controls). ePVS enlarged perivascular space, BP blood pressure, WM white matter, BG basal ganglia, HCP hippocampus, MB midbrain. Spearman Correlation coefficient values are shown. WM ePVS measurements were adjusted with the total WM value. BG, HCP, and MB ePVS measurements were adjusted with the total GM value._

**^Figure S2.^** ^Simple linear regression between BG and MB ePVS and MSNA^ **^a^** ^and^ **^b^** ^BG ePVS volume (mm3) with MSNA BF (bursts/min) and BI (bursts/100 heartbeats)^ **^c^** ^and^ **^d^** ^MB ePVS volume (mm3) with MSNA BF (bursts/min) and BI (bursts/100 heartbeats)^ **^e^** ^and^ **^f^** ^WM ePVS volume (mm3) with MSNA BF (bursts/min) and BI (bursts/100 heartbeats )^ **^g^** ^and^ **^h^** ^HP ePVS volume (mm3) with MSNA BF (bursts/min) and BI (bursts/100 heartbeats n=75 (25 hypertensive patients 50 controls)*p<0.05^

**_Table S1_** _Backward Multiple Linear Regression Analysis with Haemodynamic measures as Dependent Variable n=75_

|  | **Mean BP** | | **Systolic BP** | | **Diastolic BP** | | **Pulse Pressure** | |
| --- | --- | --- | --- | --- | --- | --- | --- | --- |
|  | ***β*** | ***p*** | ***β*** | ***p*** | ***β*** | ***p*** | ***β*** | ***p*** |
| Age (years) | - | - | - | - | - | - | - | - |
| Sex | 0.467 | <0.001 | 0.323 | <0.001 | - | - | - | - |
| Burst Incidence | - | - | - | - | - | - | - | - |
| Burst Frequency | -0.224 | 0.030 | -0.454 | 0.007 | - | - | - | - |
| Hypertension status | 0.631 | <0.001 | 0.642 | <0.001 | 0.575 | <0.001 | 0.490 | <0.001 |
| Medication status | - | - | - | - | - | - | - | - |
| WM ePVS Volume (mm^3^) | - | - | - | - | - | - | - | - |
| WM ePVS Cluster | - | - | - | - | - | - | 0.789 | 0.046 |
| BG ePVS Volume (mm^3^) | - | - | - | - | -0.287 | <0.001 | - | - |
| BG ePVS Cluster | - | - | - | - | - | - | - | - |
| HP ePVS Volume (mm^3^) | 0.240 | 0.002 | 0.183 | 0.027 | 0.159 | 0.040 | - | - |
| HP ePVS Cluster | - | - | - | - | - | - | - | - |
| MB ePVS Volume (mm^3^) | - | - | -0.168 | 0.040 | - | - | -0.426 | 0.002 |
| MB ePVS Cluster | - | - | - | - | - | - | - | - |
| TIV | - | - | - | - | - | - | - | - |
| GM | - | - | - | - | - | - | - | - |
| WM | - | - | - | - | - | - | - | - |
| Scanner | 0.631 | <0.001 | - | - | - | - | 0.277 | 0.021 |
| *Adjusted R^2^* | *0.59* |  | *0.64* |  | *0.60* |  | *0.52* |  |

_BP blood pressure, PVS perivascular space. ePVS enlarged perivascular space, WM white matter BG basal ganglia HP hippocampus TIV total intracranial volume GM grey matter_ *_β_* _standardised coefficient beta_

**_Table S2_** _Backward Multiple Linear Regression Analysis with Haemodynamic measurements as Dependent Variable n=25 hypertensives_

|  | **Mean BP** | | **Systolic BP** | | **Diastolic BP** | | **Pulse Pressure** | |
| --- | --- | --- | --- | --- | --- | --- | --- | --- |
|  | ***β*** | ***p*** | ***β*** | ***p*** | ***β*** | ***p*** | ***β*** | ***p*** |
| Age (years) | 1.340 | 0.010 | 2.107 | 0.049 | 1.297 | 0.003 | - | - |
| Sex | - | - | - | - | - | - | - | - |
| Burst Incidence | - | - | - | - | - | - | - | - |
| Burst Frequency | - | - | - | - | - | - | - | - |
| Medication use | -1.646 | 0.006 | - | - | -1.592 | 0.001 | - | - |
| WM ePVS Volume (mm^3^) | -3.021 | 0.001 | -2.550 | 0.004 | -2.715 | 0.001 | -1.617 | 0.034 |
| WM ePVS Cluster | 2.554 | 0.003 | 2.334 | 0.005 | 2.106 | 0.002 | 1.673 | 0.029 |
| BG ePVS Volume (mm^3^) | -1.376 | <0.001 | - | - | -1.151 | <0.001 | - | - |
| BG ePVS Cluster | 0.805 | 0.006 | - | - | 0.874 | <0.001 | - | - |
| HP ePVS Volume (mm^3^) | 0.602 | 0.002 | 1.304 | 0.005 | - | - | - | - |
| HP ePVS Cluster | - | - | -0.898 | 0.035 | - | - | -0.794 | 0.028 |
| MB ePVS Volume (mm^3^) | - | - | - | - | 0.589 | 0.015 | -0.754 | 0.003 |
| MB ePVS Cluster | - | - | - | - | -0.834 | 0.005 | 0.788 | 0.004 |
| TIV | - | - | - | - | 0.541 | 0.020 | - | - |
| GM | 0.450 | 0.050 | - | - | 0.343 | 0.028 | - | - |
| WM | - | - | - | - | -0.748 | 0.009 | - | - |
| Scanner | -2.204 | <0.001 | - | - | -2.271 | <0.001 | - | - |
| *Adjusted R^2^* | *0.64* |  | *0.54* |  | *0.86* |  | *0.69* |  |

_BP blood pressure, PVS perivascular space. ePVS enlarged perivascular space, WM white matter BG basal ganglia HP hippocampus TIV total intracranial volume GM grey matter_ *_β_* _standardised coefficient beta_

**_Table S3_** _Backward Multiple Linear Regression Analysis with Haemodynamic measurements as Dependent Variable n=50 normotensives_

|  | **Mean BP** | | **Systolic BP** | | **Diastolic BP** | | **Pulse Pressure** | |
| --- | --- | --- | --- | --- | --- | --- | --- | --- |
|  | ***β*** | ***p*** | ***β*** | ***p*** | ***β*** | ***p*** | ***β*** | ***p*** |
| Age (years) | 0.379 | 0.019 | - | - | - | - | -0.267 | 0.023 |
| Sex | - | - | 0.460 | <0.001 | - | - | 0.463 | <0.001 |
| Burst Incidence | 0.734 | 0.024 | - | - | - | - | - | - |
| Burst Frequency | -0.792 | 0.014 | -0.329 | 0.009 | - | - | - | - |
| WM ePVS Volume (mm^3^) | - | - | - | - | - | - | - | - |
| WM ePVS Cluster | - | - | - | - | - | - | - | - |
| BG ePVS Volume (mm^3^) | - | - | -0.343 | 0.016 | - | - | - | - |
| BG ePVS Cluster | - | - | - | - | - | - | - | - |
| HP ePVS Volume (mm^3^) | - | - | - | - | - | - | - | - |
| HP ePVS Cluster | - | - | - | - | - | - | - | - |
| MB ePVS Volume (mm^3^) | - | - | - | - | - | - | - | - |
| MB ePVS Cluster | - | - | - | - | - | - | - | - |
| TIV | - | - | -0.780 | 0.020 | - | - | - | - |
| GM | - | - | - | - | - | - | - | - |
| WM | - | - | 0.779 | 0.016 | - | - | - | - |
| Scanner | - | - | - | - | -0.516 | <0.001 | 0.280 | 0.039 |
| *Adjusted R^2^* | *0.19* |  | *0.40* |  | *0.25* |  | *0.60* |  |

_BP blood pressure, PVS perivascular space. ePVS enlarged perivascular space, WM white matter BG basal ganglia HP hippocampus TIV total intracranial volume GM grey matter_ *_β_* _standardised coefficient beta_

**_Table S4._** _Participant demographics and medication information for hypertensive patients_

| **Age** | **Sex** | **Scanner** | **Medication** | **Duration of hypertension** | **BMI** | **OSA (yes/no)** | **Diabetes Mellitus (yes/no)** | **Renal dysfunction (y/n)** | **Other diseases (yes/no)** |
| --- | --- | --- | --- | --- | --- | --- | --- | --- | --- |
| 70 | male | Siemens | Zanidip 20mg, Micardis Plus 80/25mg, Metoprolol 50mg, Ezetrol 10mg, Cartia 100mg, Duodart 500/400 mcg, Vitamin B12 Tablets, Physiotens tablets 0.4mg, Spiractin tablets 25mg, Saxenda solution in injection 6mg/1ml, 3ml | 11 years + (2007 renal denervation) | N/A | N | N | N | Y (Resistant hypertension, bilateral renal artery denervation 2007, previous TIA, obesity, pure hypercholesterolaemia, sleep hypoventilation syndrome, Bell’s palsy, raised fasting glucose (IFG), sarcoidosis of lymph nodes) |
| 75 | female | Siemens | Coloxyl with Senna, Flixotide 125μgm, Ostelin 1000IU, Telmisartan 40mg, Hydrochlorothiazide 12.5mg, Rosuvastatin 5mg | Very long standing (>20 years; retinal and fundal abnormalities with HMOD) | 26.96683224 | N | N | Y | Y(COPD) |
| 46 | male | Siemens | Peripheral acting antihypertensive medication | N/A | 32.95 | N | N | N | N |
| 61 | male | Siemens | Methyldopa, Angiotensin II Receptor Blocker, Calcium channel bloker, Alpha Blocker, Spironolactone | Long standing (>20 years) (2013 renal denervation) | 34.89 | N | N | Y (eGFR <40) | N |
| 47 | female | Siemens | Telmisartan 40mg | Dx in 2008; 11 years | 20.36 | N | N | N | N |
| 58 | male | Siemens | Twynsta, Ramipril | 7 months (Dx on 18/6/2018) | 23.6 | N | N | N | N |
| 36 | male | Siemens | Peripheral acting antihypertensive medication | N/A | 24.54 | N | N | N | N |
| 57 | female | Siemens | Peripheral acting antihypertensive medication | N/A | 30.24 | N | N | N | N |
| 79 | male | Siemens | Peripheral acting antihypertensive medication | N/A | 24.13 | N | N | N | N |
| 57 | male | Siemens | Peripheral acting antihypertensive medication | N/A | 28.7 | N | N | N | Y(Hypercholesterolemia) |
| 25 | male | Siemens | Undiagnosed | Unknown | 24.3 | N | N | N | N |
| 21 | male | Philips | Undiagnosed | Unknown | N/A | N | N | N | N |
| 20 | male | Philips | Undiagnosed | Unknown | N/A | N | N | N | N |
| 19 | male | Philips | Undiagnosed | Unknown | N/A | N | N | N | N |
| 22 | male | Philips | Undiagnosed | Unknown | N/A | N | N | N | N |
| 26 | male | Philips | Undiagnosed | Unknown | N/A | N | N | N | N |
| 21 | male | Philips | Undiagnosed | Unknown | N/A | N | N | N | N |
| 21 | male | Philips | Undiagnosed | Unknown | N/A | N | N | N | N |
| 19 | male | Philips | Undiagnosed | Unknown | N/A | N | N | N | N |
| 27 | male | Philips | Undiagnosed | Unknown | N/A | N | N | N | N |
| 21 | male | Philips | Undiagnosed | Unknown | N/A | N | N | N | N |
| 28 | female | Philips | Undiagnosed | Unknown | N/A | N | N | N | N |
| 34 | male | Philips | Undiagnosed | Unknown | N/A | N | N | N | N |
| 61 | male | Siemens | Ramipril | Unknown | 25.6 | N | N | N | N |
| 69 | female | Siemens | Irbesartan (150mg) | 10 years | 34.1 | N | Y (Type 2) | N | N |
